# Breaking Abbe’s diffraction limit with harmonic deactivation microscopy

**DOI:** 10.1126/sciadv.adp3056

**Published:** 2024-11-13

**Authors:** Kevin Murzyn, Maarten L. S. van der Geest, Leo Guery, Zhonghui Nie, Pieter van Essen, Stefan Witte, Peter M. Kraus

**Affiliations:** ^1^ Advanced Research Center for Nanolithography (ARCNL), Science Park 106, 1098 XG Amsterdam, Netherlands.; ^2^ Department of Physics and Astronomy, and LaserLaB, Vrije Universiteit, De Boelelaan 1081, 1081HV Amsterdam, Netherlands.

## Abstract

Nonlinear optical microscopy provides elegant means for label-free imaging of biological samples and condensed matter systems. The widespread areas of application could even be increased if resolution was improved, which the famous Abbe diffraction limit now restrains. Super-resolution techniques can break the diffraction limit but most rely on fluorescent labeling. This makes them incompatible with (sub)femtosecond temporal resolution and applications that demand the absence of labeling. Here, we introduce harmonic deactivation microscopy (HADES) for breaking the diffraction limit in nonfluorescent samples. By controlling the harmonic generation process on the quantum level with a second donut-shaped pulse, we confine the third-harmonic generation to three times below the original focus size of a scanning microscope. We demonstrate that resolution improvement by deactivation is more efficient for higher harmonic orders and only limited by the maximum applicable deactivation-pulse fluence. This provides a route toward sub-100-nanometer resolution in a regular nonlinear microscope.

## Teaser

Controlling high harmonic emission in space and time enables resolution below the diffraction limit.

## INTRODUCTION

Far-field optical microscopy has affected the fields of medicine, biology, and biophysics (*1*) like hardly any other technique. Microscopy is used as a daily diagnostic tool as well as equipment for cutting-edge scientific advancements. Microscopy benefits from its compactness, ease of use, and the possibility to image two-dimensional (2D) as well as 3D objects (*2*). Third-harmonic generation (THG) is used as a label-free microscopy (*3*) method to study cells (*4*, *5*), photoresists (*6*), and 2D materials (*7*). For THG microscopy, an excitation pulse is focused into a material and generates harmonics. The high penetration depth of the near-infrared (NIR) excitation light and the sensitivity to changes in chemical composition are central advantages of THG compared to other optical contrast mechanisms. One challenge preventing an even more widespread application is the limited resolution. The reported resolution of THG microscopes is approximately 1 μm or slightly less (*8*, *9*), which is far from the 200-nm resolution limit commonly stated in a confocal fluorescence microscope (*10*). This apparent limitation stems from the fact that in typical confocal scanning microscopes, the resolution is proportional to the focus size of the excitation laser of the emission process. This laser is typically in the infrared for harmonic microscopy but in the visible or ultraviolet range for fluorescent microscopy. To improve this resolution limit of harmonic microscopy, the emission has to be confined below the diffraction limit. Here, we show that the unique optical properties of high-harmonic generation (HHG) from condensed matter enable subdiffraction harmonic emission and thus super-resolution microscopy.

HHG is a laser-driven frequency upconversion process (*11*, *12*), which converts an ultrashort laser pulse into a shorter-wavelength pulse at integer multiples of the fundamental carrier frequency. The discovery of solid-state HHG in 2010 (*13*) also rejuvenated the interest in harmonic generation of lower orders below the bandgap (*14*) and drove the development of a stringent microscopic theoretical framework (*15*, *16*) that complements the phenomenological framework of perturbative nonlinear optics. On the quantum level, HHG is governed by laser-driven electron dynamics and contains signatures of the dynamic electronic structure of the generation medium that can be atoms (*17*), molecules (*18*, *19*), and solids (*13*, *20*). Inversely, slight variations to these electronic dynamics can have a large impact on the efficiency of HHG (*21*). In a variety of solids, reversible deactivation of high harmonics followed the photoexcitation of charge carriers (*22*–*27*). Different physical processes are considered to contribute to the deactivation (*28*, *29*) such as state-blocking and ground-state depletion, a decrease in decoherence time (*26*, *27*, *30*), and control of electron-hole recollisions in shaped driving waveforms (*31*). The modulation depth, or contrast, achieved can be close to 100%, demonstrating near–unity-contrast emission control. This intensity modulation is the key enabler of super-resolution HHG microscopy by spatially confining HHG with an optical control pulse.

We took inspiration from the super-resolution techniques in fluorescence microscopy, which can provide resolutions below 10 nm (*32*). These techniques can be divided into two groups. The first group uses wide-field illumination of the sample and achieves super-resolution through stochastic on-and-off switching of fluorescence combined with localization algorithms (*33*–*36*). The second group consists of deterministic methods that rely on targeted modulation or saturation of fluorescence (*37*–*40*). Here, we use the high modulation depth of HHG (*41*) for an approach similar to the one used in stimulated emission depletion (STED) microscopy (*38*) for HHG microscopy. We label this method as harmonic deactivation microscopy (HADES), which relies on an increase in incoherent scattering and disruption of the recombination process through a control beam. In the present study, we focus on THG, but the method will work similarly and with even better resolution for higher harmonic orders. In HADES, we add a donut-shaped deactivation pulse to the infrared driving pulse of HHG. This donut pulse deactivates harmonic emission in the outer parts of the point-spread function (PSF) of the excitation pulse leading to a narrower PSF compared to THG. A similar methodology has been shown for linear microscopy in silicon (*42*) and graphene (*43*), relying on the plasma dispersion effect and absorption saturation, respectively. The latter hereby was able to achieve a resolution of below 200 nm. For nonlinear microscopy using coherent anti-Raman scattering, methods were patented (*44*) and later shown experimentally (*45*). Improvements of the resolution of second-harmonic generation microscopy have been shown previously through coherent rescanning (*46*) and exploiting the nonlinear interaction in a structured illumination microscope (*47*). A twofold resolution improvement is achieved through controlling the polarization state over the focus spot (*48*). In this work, we demonstrate a 2D resolution improvement using increased incoherent scattering and recombination during the HHG process.

The combination of ultrafast temporal and nanoscale resolution will be a unique advantage of HADES: Because of the necessity of intramolecular vibrational energy redistribution before stimulated emission in STED, fluorescence super-resolution is inherently incompatible with femtosecond time resolution (*49*).

While deactivation is a general phenomenon, we chose NbO_2_ as a sample in the present manuscript. NbO_2_ is a strongly correlated material that undergoes a nonthermal ultrafast insulator-to-metal phase transition (IMT) upon photoexcitation. This phase transition makes strongly correlated materials interesting for a new class of electronic devices such as memristors (*50*, *51*). In recent work, HHG was used to probe this transition in NbO_2_ and measure its transition timescale to be ~100 fs (*41*). The ability to add super-resolution to resolving IMTs will enable resolving the emergence of different phases with femtosecond temporal and nanometer spatial resolution.

In this letter, we show the time-dependent deactivation behavior of THG in NbO_2_ and how it is governed by the fluence of the deactivation beam. These results are used to predict a change in the PSF of the third harmonic in HADES. We then change in our experiment the deactivation beam profile to a donut shape and confirm the narrower PSF. Conclusively, we improve the resolution of a scanning THG microscope by implementing HADES.

## RESULTS

### Deactivation of THG

**Fig. 1.**
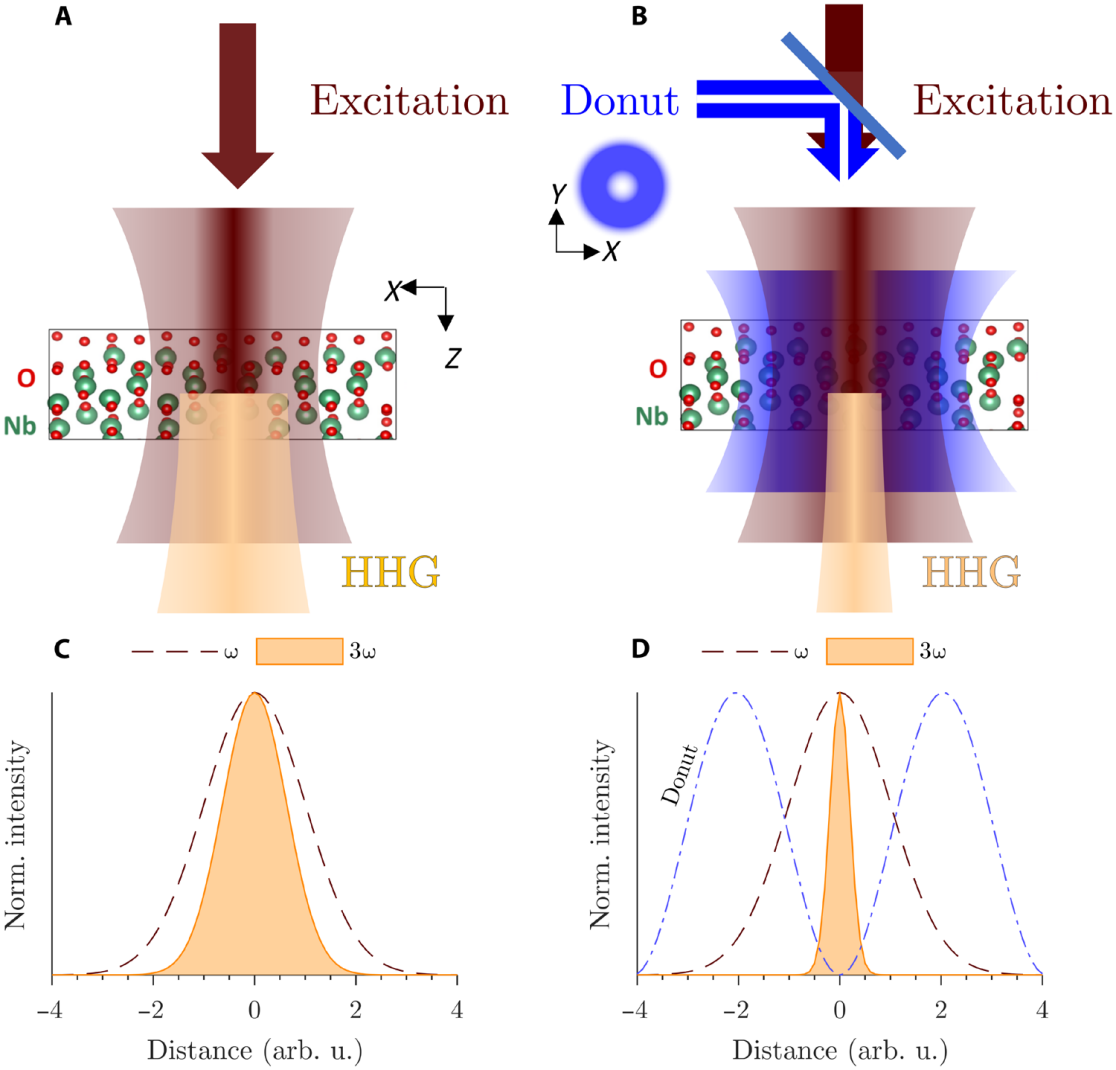
Principle of HADES. Schematic of the illumination scheme (**A** and **C**) and normalized intensities in the focal spot (**B** and **D**) for HHG microscopy [(A) and (C)] and for HADES [(B) and (D)]. In an HHG microscope, a fundamental laser pulse at central frequency ω excites HHG. Compared to this, an additional donut-shaped pulse deactivates high harmonic emission outside of the center. The resulting spot size which effectively can be observed is reduced substantially.

The concept of the experiment is illustrated in Fig. 1. In regular harmonic microscopy, a NIR excitation laser generates harmonic multiples of its fundamental frequency. For a Gaussian intensity distribution *I*_NIR_ of the fundamental NIR excitation laser in focus (Fig. 1A), the intensity distribution of the third harmonic will also be Gaussian (Fig. 1C). The spatial emission intensity distribution, called PSF, of HHG is narrower than the excitation pulse PSF and scales in the perturbative regime with *I*_HHG_ ∝ $I_{\text{NIR}}^{n}$, where *n* is the power law exponent of the process which is approximately three for THG. In this work, we further deactivate HHG spatially by adding a pulse that is donut shaped in focus to HHG (Fig. 1B). This donut-shaped pulse is achieved by imparting an orbital angular momentum onto the deactivation pulse by means of a spiral phase plate inserted in the far-field pulse profile. This concept allows deactivating HHG to a narrower emission peak below the diffraction limit (Fig. 1D) since HHG is deactivated in regions of nonzero intensity of the donut-shaped deactivation pulse. By narrowing the spot size below the diffraction limit, we can implement a super-resolution label-free scanning microscope.

We use a modified Mach-Zehnder interferometer to realize these experiments (fig. S1). The fundamental HHG driving pulse is centered at a wavelength of 1830 nm, while the donut pulse is centered at 400 nm. Both pulses are linearly and parallel polarized. Detailed descriptions of all experiments can be found in Materials and Methods.

**Fig. 2.**
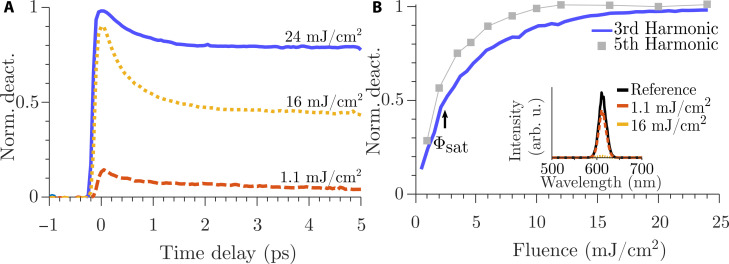
Deactivation mechanism. (**A**) Time-dependent normalized deactivation of the third harmonic response plotted at rising fluences of the modulation pulse. At negative times, the NIR excitation pulse arrives first. The strongest deactivation is always seen at the temporal overlap of the two pulses. (**B**) Fluence-dependent deactivation of the third harmonic (blue) and fifth harmonic [gray; taken from (*41*)] normalized to the reference harmonic emission without modulation pulse. The saturation fluence ϕ_sat_ is defined as the point of 50% normalized deactivation. The fifth harmonic shows steeper deactivation and potentially better resolution improvement. The inset shows the third harmonic spectra of the reference (blue) and the spectra with a modulation fluence of 1 mJ/cm^2^ (orange) and a modulation fluence of 16 mJ/cm^2^ (yellow).

We first characterize the deactivation behavior by measuring the spectra of the emitted harmonics. We vary the delay between the pulses (Fig. 2A) and the intensity of the deactivation pulse (Fig. 2B). The spectral shape of the third harmonic (Fig. 2B, inset) stays identical for all fluences. However, with increasing modulation fluence, the amplitude and the integrated signal reduce. We normalize the integrated signal to its deactivation, i.e., a normalized deactivation of one means full deactivation of HHG so that there is no measurable signal, while zero means the signal is unchanged. When the driving pulse arrives before the deactivation pulse, no change in the THG can be observed. This is followed by a sharp increase in the maximum deactivation when both pulses arrive simultaneously. At increasing time delays, the signal recovers with a fast and slow exponential component, consistent with the results in (*41*).

The deactivation becomes stronger the higher the deactivation-pulse fluence. However, no dependence on the relative polarization angle could be observed during the experiments. At the highest deactivation-pulse fluence of 24 mJ/cm^2^, the integrated signal amounts to 2% of the reference at the temporal overlap of the two pulses. From this fluence dependence of the normalized deactivation, we determine a saturation fluence Φ_sat_, defined as the fluence at half deactivation, of 2.5 mJ/cm^2^, while the intensity modulation depth is 98%. Comparing these results to deactivation curves for the fifth harmonic (Fig. 2B, gray curve) shows that higher-order harmonics have a lower saturation fluence of 1.8 mJ/cm^2^.

While other STED-like techniques have been proposed and implemented in the past (*45*, *48*), our approach—HADES—sets itself apart, both in its physical mechanism and its prospect for broad applicability. These intertwined features—broad applicability through a new mechanism—rest on how solid HHG can be deactivated by a modulation pulse, which is first and foremost a property of the HHG process on the quantum level as well as the driving field but not of the material. The mechanistic understanding of solid harmonic generation has been rejuvenated in recent years (*20*) and extended beyond the phenomenological perturbative nonlinear picture. Solid HHG is understood to originate from two contributions following multiphoton excitation across the bandgap. HHG is emitted from an intraband carrier current, as well as an interband polarization due to coherent electron-hole pairs. This interband polarization can be thought to originate from an electron-hole recollision. If a second modulation pulse is applied to control the HHG process, several phenomena occur as detailed in our recent perspective article on the topic (*28*) and briefly reviewed here: First, two-color waveforms strongly modulate HHG (*52*, *53*), and the waveform of two overlapping electric fields (i.e., at a time delay of zero) with incommensurate frequencies can decrease the overall intensity within a harmonic order (*54*). Second, right after excitation and for later times (i.e., for delays of and larger than zero), the coherent electron-hole pairs excited from the HHG driving pulse dephase faster due to the excited carrier population from the first pulse. In general, an excited carrier population promotes both accelerated carrier-carrier and carrier-phonon scattering, thus leading to a rapid loss of electron-hole coherence (*23*, *27*). It is this electron-hole coherence which is neccessary for efficient HHG, so an increased dephasing causes an amplitude reduction and thus deactivation of HHG. In the specific case of NbO_2_, photoexcitation triggers an ultrafast phase transition on a timescale of 100 fs (*41*) into a metallic phase, where electron scattering is generally larger and thus the effect of HHG suppression through loss of electron-hole coherence is further enhanced. Scattering and the associated dephasing-time reduction act as amplitude suppression (*41*) window onto the time-domain emission of HHG, thus reducing the overall emission intensity. Both effects, waveform modulation and increased electron dephasing, are contributing to deactivation in our experiment. This is evidenced by the observation of the highest levels of HHG deactivation at time zero for all pump fluences, which can thus be partially attributed to waveform modulation. Furthermore, the persisting high level of HHG deactivation after time zero can be attributed to increased scattering due to excited electrons and also an IMT at higher pump fluences. At a time delay of zero, where the experiments described in this article are performed, both effects can thus play a role. Critically, none of these processes rely on resonant transitions in the material itself as the harmonic deactivation has been shown also for below bandgap pumping (*28*, *31*, *55*).

### PSF reduction

We first estimate the expected resolution improvement of HADES for a 1D model. We combine the fluence-dependent deactivation with the spatial distribution of the intensity of the donut pulse and multiply this with an initial Gaussian distribution of the emitted harmonics. Assuming a maximum fluence of 25 mJ/cm^2^, this 1D model results in a PSF reduction by a factor of three (fig. S2). This requires that the donut-shaped pulse has a constant azimuthal fluence distribution and that the maximum of the donut coincides with the full width at half maximum (FWHM) of the THG, since a larger donut pulse lessens the effective applied fluence and a smaller donut pulse produces side wings which lead to smearing.

In the above model, we chose to use a cos^2^ function for modeling the donut-shaped deactivation profile as previously done in (*56*) for STED microscopy. We, therefore, modify the resulting formula to be adapted for our measurements as (*56*)

$$d=\frac{\lambda_{0}}{2\text{NA}\sqrt{n}}\frac{1}{\sqrt{1+\zeta }}$$ (1)

The first term containing the wavelength of the fundamental excitation laser λ_0_, nonlinear order *n* (three or five for detection of the third or fifth harmonic, respectively, in the perturbative regime), and numerical aperture NA is the diffraction limit after Abbe modified for harmonic microscopy. The second term gives the resolution improvement based on the saturation level $\zeta =\Phi_{\text{HADES}}^{\text{peak}}/\Phi_{\text{sat}}$. If we assume a peak fluence $\Phi_{\text{HADES}}^{\text{peak}}$ of 24 mJ/cm^2^ and the measured saturation fluence for the third harmonic, the resolution improves by a factor of 3.3, which is close to our 1D model. For fifth-harmonic generation, we calculate a resolution improvement of 3.8. This shows pathways to improve the resolution even further by decreasing the saturation fluence, e.g., via optimizing the deactivation scheme or by using higher-order harmonics that deactivate at lower fluences.

**Fig. 3.**
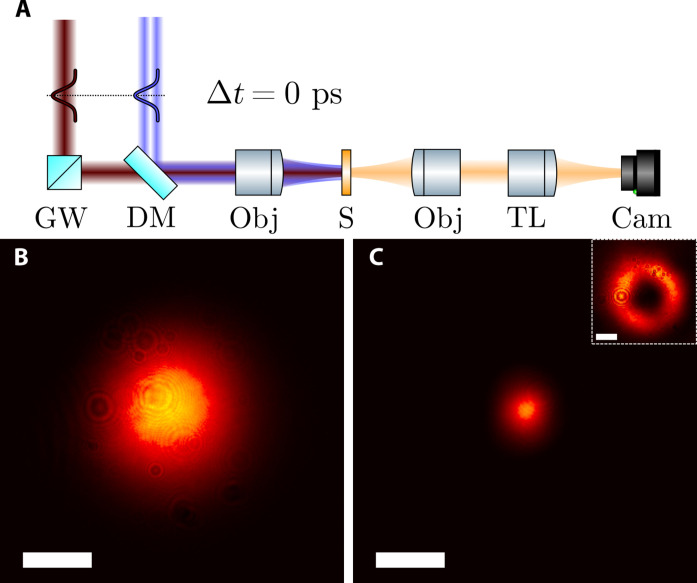
PSF reduction with donut pulse. The excitation pulse (red) and donut pulse (blue) get combined and focused onto the sample by a lens with a 30-cm focal length. A home-built microscope images the PSF of the transmitted harmonics (**A**). PSF on the sample without (**B**) and with (**C**) the OAM pulse, the inset shows the deactivation pulse. The bar is 30 μm, which is the resolution limit of the focusing objective at the THG wavelength. GW, glass wedge; DM, dichroic mirror; Obj, objective lens; S, sample; TL, tube lens; Cam, camera.

We now demonstrate this all-optical control and shrinkage of the PSF. A lens with a focal length of 25 cm generates the third harmonic in the sample (Fig. 3A). A home-built bright-field microscope images the PSF in transmission. We calculate the diffraction limit of the lens that focuses the driving pulses as 40.7 ± 1.2 μm, which is the first term in Eq. 1. The unchanged third harmonic PSF (Fig. 3B) has an FWHM of 40 ± 1.5 μm, which is in agreement with the theoretical diffraction limit. When the donut pulse (Fig. 2C, inset) deactivates the third harmonic emission, the area that emits light is drastically reduced to an FWHM of 14 ± 0.6 μm, while the peak intensity stays similar. This yields a reduction of factor 2.9 ± 0.15, which matches the prediction of the 1D model. On the basis of the experimental data, we calculated a 2D model, which also shows a PSF reduction although this is limited by the diffraction from the uneven sample surface (fig S3). The emission spot produced via HADES is therefore roughly three times smaller than Abbe’s diffraction limit in Eq. 1. This paves the way toward a feasible approach for label-free super-resolution scanning microscopy through HADES, which we demonstrate in the next section in a proof-of-principle experiment.

### Microscopic imaging

**Fig. 4.**
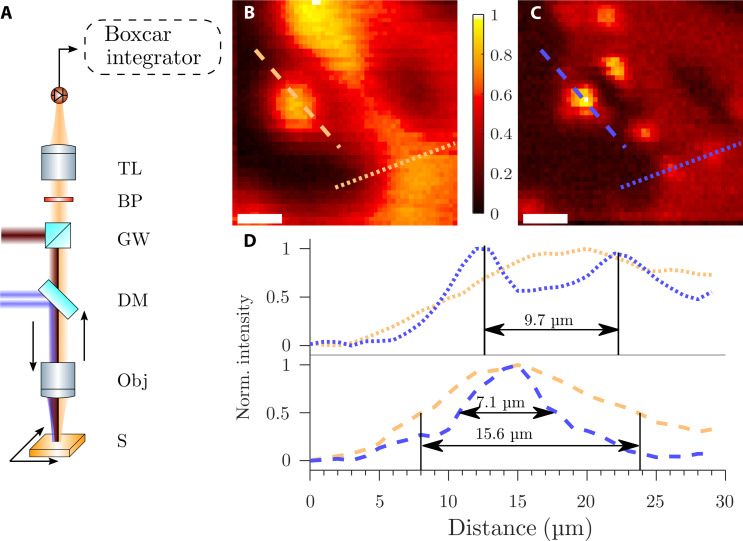
Resolution improvement through HADES. An NIR (red) and NIR donut-shaped (blue) pulse are focused onto the sample (**A**). The back-reflected third-harmonics (orange) are detected by an APD. Scanning the sample through the focus spot generates the image. Blocking the donut pulse results in a THG image (**B**). Adding the donut pulse (**C**) gives rise to an image through HADES. The color bar applies to both images and shows the normalized intensity. Scale bars, 10 μm. The bottom (**D**) shows profiles along the dotted line and the dashed line in the above pictures. The profiles in blue are taken from the THG microscopy picture and in orange from the HADES picture. These profiles show a notable resolution improvement for HADES over THG microscopy. BP, bandpass filter.

For rapid sampling, the fundamental pulse of 800 nm was used for deactivation since a similar deactivation behavior to 400 nm was observed. We used a lens with a focal length of 5 cm as the focusing objective, which also captured the back-reflected THG for imaging (Fig. 4A). In the detection pathway, a series of bandpass filters selects a specific harmonic order. A tube lens then focuses the filtered light onto an avalanche photodiode (APD). The APD measures signal with low average powers on a shot-to-shot basis, enabling rapid scanning of the sample. A boxcar integrator filters out noise outside the 2% duty cycle. Figure 4 (B and C) shows the recorded pictures for THG microscopy and HADES, respectively. These pictures depict the transition from an area with laser-induced damage to an undamaged sample area. The large dark area (area with no signal) has no thin film left, while the area emitting a lot of signal (red area) still has thin film. We analyze the origin of the sharp intensity features in Discussion. In comparison, the HADES picture visualizes smaller details compared to THG microscopy. The super-resolution becomes visible when convolving the HADES image with a Gaussian. This fully reproduces the THG microscopy image (fig. S4), which demonstrates the resolution improvement by HADES. The two profiles (Fig. 4D) exemplify this resolution improvement further. Along the dotted line in the THG microscope, a single peak is imaged while by using HADES two peaks become distinguishable. These two peaks are separated by 9.7 μm. The dotted line depicts a single peak at the very edge of the thin film. With THG microscopy the FWHM of this emitter is 15.6 μm, while with HADES, the FWHM reduces to 7.1 μm. These examples can be quantified further by the use of a Fourier ring correlation (FRC) (*57*) analysis. First, a mask was applied to the Fourier transform of the images to suppress scanning artifacts. The FRC shows a higher correlation at larger spatial frequencies for HADES (fig. S5). We choose the ^1^/_7_ threshold (*58*), as it is a common threshold for resolution estimation in 2D. This threshold yields a resolution limit of 8.6 and 5.5 μm for THG and HADES, respectively. We show, therefore, that the resolution improves in two dimensions at the same time.

## DISCUSSION

We now turn to discussing signal formation in HADES. The image shows an area of the NbO_2_ thin film that was thermally damaged before the HADES experiments. In bright-field microscopy, the dark area is visible as well, indicating that material was ablated (fig. S6A) when damage occurred. The bright spot (dashed profile in Fig. 4) is located at a pointy tip of the thin film to pure substrate transition seen in dark-field microscopy (fig. S6B). A combination of multiple effects can be the reason for the enhancement of the THG signal at this bright spot. First, the third-order susceptibility of the sample can be changed locally, since the NbO_2_ thin film generates harmonics more efficiently than the pure substrate. Second, near-field confinements of the electric field of the fundamental in subwavelength nanostructures can enhance the HHG efficiency (*59*). The presence of such subwavelength nanostructures becomes visible under a scanning electron microscope (fig. S7). During experimenting, we generally observed an enhancement of the signal when scanning over an edge in the sample, indicating near-field enhancement of the fundamental field, which boosts the efficiency of HHG locally.

Our current experimental procedure was challenged by the available low repetition rate (2 kHz)/high-pulse energy (3.5 mJ) laser system. The high pulse energies required a strong attenuation of both the modulation and excitation pulse by effective optical densities up to 5, which made detectable signals weak due to the 2-kHz repetition rate. These requirements made optimization more challenging especially for shorter focal lengths lenses. Furthermore, scanning microscopy as shown in Fig. 4 will benefit from either high-speed scanning stages or ideally a galvo-mirror system that scans the position of the focus through the objective, neither of which was available for the current experiment. Implementing both higher repetition rate lasers and high-speed scanning will make taking microscopy images in seconds possible. This will also allow focusing the HHG driving pulses with high-NA objectives instead of lenses (Fig. 4). Since we have demonstrated the mechanism for breaking the diffraction limit in harmonic microscopy in this manuscript, we now understand that we can quantitatively predict the resolution improvement by means of Eq. 1 and the harmonic deactivation curve (Fig. 2B).

The ideas presented can be extended to tighter focusing conditions emitting higher harmonics. The in-depth analysis of the deactivation process measured here and in (*41*) delivers a three times resolution improvement of THG microscopy and a 3.8 times resolution improvement for a fifth-harmonic generation microscope. Considering an NIR excitation at 1800 nm and a high NA objective of 1.35, the expected resolutions for the specific set of wavelengths, polarizations, and material (NbO_2_) chosen here are 120 nm for THG and 80 nm for the fifth-harmonic generation. These estimations are made on the basis of Eq. 1, and the experimental findings done in this paper and in (*41*), which yields a saturation fluence of 1.8 mJ/cm^2^ for the fifth harmonic. The maximum applicable fluence was hereby always low enough to not damage the sample itself, meaning that laser-induced damage ultimately limits this technique. Deepening the understanding of the deactivation process for harmonic generation in materials will lead to an even better resolution by either reducing the saturation fluence, i.e., finding ways to deactivate HHG more efficiently, or by extending the highest applicable peak fluence. This principle is ultimately not limited to the deactivation of HHG by photoexcited carriers, which limits the maximum applicable fluence due to photo-induced damage. Other deactivation methods are also suited for this, i.e., control of the electron-hole recombination with a combination of below bandgap driving wavelengths might also be a suitable pathway (*31*). Ultimately, resolution in HADES is only limited by the real-space excursion of electrons and holes by the driving laser pulse. This excursion underlies the microscopic currents that radiate HHG and corresponds to a real-space displacement on the single-digit nanometer scale, the exact value of which depends on electronic structure and laser parameters. As a hypothetical deactivation below this limit would truncate HHG even from the inner region without a deactivation pulse, this excursion length scale sets the ultimate resolution limit for HADES. Furthermore, other optical nonlinearities such as coherent anti–Stokes Raman scattering (*60*), stimulated Raman scattering (*61*), or sum frequency generation (*62*) can be used to achieve super-resolution with similar concepts. However, the extreme nonlinearity of HHG creates a maximum sensitivity to control pulses. This likely makes HHG and related high-order frequency mixing processes the ideal candidates for resolution improvement by deactivation in particular and optical emission control and optical switching in general. The wide range of solid-state materials, which have shown harmonic deactivation (*28*), makes HADES an interesting candidate for metrology on dielectric and semiconducting materials, as well as an important research tool for material science. Purley field effect-driven harmonic deactivation allows even application in biological samples.

## MATERIALS AND METHODS

### Materials

The sample investigated in this study is the polycrystalline thin-film NbO2, which was grown on a c-plane sapphire substrate through reactive bias target ion pulse deposition, and the more information of the material synthesis is available in previous literature (*41*, *63*). The Raman spectrum and x-ray diffraction 2θ scan are both used to confirm the high quality and the body-centered tetragonal (bct) lattice structure of our sample. Any other oxide components (NbO or Nb_2_O_5_) have not been found. The thickness of our sample is ∼115 nm, measured by x-ray reflectivity, and its surface roughness is less than 1 nm. Moreover, the sapphire substrates are double-side polished, ensuring the possibility of transmission measurements.

### Methods

#### Laser system and experimental setup

A Ti:Sapphire laser amplifier (Solstice, Spectra-Physics) produces a laser pulse at a central wavelength of 790 nm and with a pulse duration below 72 fs. The repetition rate is set to 2 kHz, while the average output power is 7 W. A beam splitter (BS) sends the laser light into each of the arms. In the excitation arm, the light pumps an optical parametric amplifier (TOPAS Prime), which emits NIR light centered at 1800 nm. The idler of the parametric process was used to generate the third harmonic since only the idler can reach the desired wavelength. In the modulation arm, the delay of the 800 nm pulse can be adjusted by a mechanical delay stage. This is called the modulating arm. Afterward, a spiral phase plate converts the intensity profile into a donut shape. Optionally, a second-harmonic generation BBO can be introduced after the spiral phase plate to change the central wavelength to 400 nm. Variable attenuators control the average power in each arm independently, while telescopes set the pulse size. A dichroic mirror combines the two pulses. After this, an objective lens focuses this pulse bundle onto the sample. The modulation arm uses a combination of a half-wave plate and polarizer for a first attenuation, and a second attenuation is achieved by a variable neutral density filter wheel. The excitation arm is attenuated by a series of neutral density filters. A schematic of the experiment can be found in the Supplementary Materials (fig. S1).

#### Deactivation measurements

For the deactivation measurements, we removed the spiral phase plate from the modulation arm. A telescope focuses the modulation pulse so that it has an FWHM of 200 μm, while the excitation NIR pulse has an FWHM of 40 μm. A tube lens focuses the back-reflected harmonics into a fiber spectrometer (Ocean Insight, QE Pro), that resolves the spectral composition of the harmonics.

#### PSF imaging

A microscope objective (Mitutoyo Plan Apo NIR B) collimates the light from the sample with a magnification of ×20 and an NA of 0.4. Subsequently, a tube lens with a focal length of 20 cm images them onto a camera. A pair of Bandpass filters blocked the donut pulse for PSF imaging. They have a central wavelength of 600 nm and a FWHM of 40 nm. The donut pulse was attenuated by an neutral-density filter with an optical density of 4.0 to avoid permanent damage. We evaluated the FWHM of the PSF by binarizing the images at a relative intensity of 0.5. Then, an ellipse was fitted to the mask created that way. The FWHM represents the average radius of the fitted ellipse.

#### Microscopy pictures

For the microscopy images, both arms had to be attenuated by an effective optical density of 5. Otherwise, the high pulse energy of the laser could damage the thin-film surface. An 800-nm pulse deactivated the third harmonic response. To make scanning images, we used a lens with a focal length of 5 cm and detected the reflected third harmonic emission. A silicon-based analog APD (Thorlabs) produces the signal out of the THG. A preamplifier amplified the voltage output of the APD before the pulsed signal was integrated using a boxcar integrator. The voltage output was then digitized by a data acquisition card. A piezo stage moved the sample through the focused pulse from spot to spot.

The images have a pixel size of 1 μm in both directions and are 51 × 51 pixels large. The images were scanned line by line. For the THG microscopy at each pixel, 25 laser pulses were measured, and each line was repeated three times, while for HADES, each line was repeated 12 times with 25 laser pulses per pixel to ensure a similar count of photons for each pixel. The microscopy pictures displayed are the averaged values of each measurement, normalized to the maximum and minimum values. For the profile generation, the images were smoothed using a median filter with a pixel radius of 1.5. The profiles were generated with the profile plotter of ImageJ. The profiles were again normalized and interpolated by a spline algorithm for measurements of the FWHM of the features and measurements of the peak separation.

## References

[1] S. Weisenburger, V. Sandoghdar, Light microscopy: An ongoing contemporary revolution. Contem. Phys. 56, 123–143 (2015).

[2] J. Mertz, *Introduction to optical microscopy* (Cambridge Univ. Press, 2019).

[3] M. D. Duncan, J. Reintjes, T. J. Manuccia, Scanning coherent anti-stokes raman microscope. Opt. Lett. 7, 350–352 (1982). 19714017 10.1364/ol.7.000350

[4] D. Débarre, W. Supatto, A. M. Pena, A. Fabre, T. Tordjmann, L. Combettes, M. C. Schanne-Klein, E. Beaurepaire, Imaging lipid bodies in cells and tissues using third-harmonic generation microscopy. Nat. Methods 3, 47–53 (2006). 16369553 10.1038/nmeth813

[5] S. Witte, A. Negrean, J. C. Lodder, C. P. De Kock, G. T. Silva, H. D. Mansvelder, M. L. Groot, Label-free live brain imaging and targeted patching with third-harmonic generation microscopy. Proc. Natl. Acad. Sci. U.S.A. 108, 5970–5975 (2011). 21444784 10.1073/pnas.1018743108 PMC3076839

[6] L. Kallioniemi, S. Annurakshita, G. Bautista, Third-harmonic generation microscopy of undeveloped photopolymerized structures. OSA Contin. 3, 2961 (2020).

[7] L. Zhou, H. Fu, T. Lv, C. Wang, H. Gao, D. Li, L. Deng, W. Xiong, Nonlinear optical characterization of 2D materials. Nanomaterials 10, 2263 (2020). 33207552 10.3390/nano10112263 PMC7696749

[8] M. Yildirim, N. Durr, A. Ben-Yakar, Tripling the maximum imaging depth with third-harmonic generation microscopy. J. Biomed. Opt. 20, 096013 (2015). 26376941 10.1117/1.JBO.20.9.096013

[9] Y. Murakami, M. Masaki, S. Miyazaki, R. Oketani, Y. Hayashi, M. Yanagisawa, S. Honjoh, H. Kano, Spectroscopic second and third harmonic generation microscopy using a femtosecond laser source in the third near-infrared (NIR-III) optical window. Biomed. Opt. Express 13, 694–708 (2022). 35284173 10.1364/BOE.446273 PMC8884214

[10] L. Shao, P. Kner, E. H. Rego, M. G. Gustafsson, Super-resolution 3D microscopy of live whole cells using structured illumination. Nat. Methods 8, 1044–1046 (2011). 22002026 10.1038/nmeth.1734

[11] A. McPherson, G. Gibson, H. Jara, U. Johann, T. S. Luk, I. McIntyre, K. Boyer, C. K. Rhodes, Studies of multiphoton production of vacuum-ultraviolet radiation in the rare gases. J. Opt. Soc. Am. B 4, 595–601 (1987).

[12] M. Ferray, A. L’Huillier, X. F. Li, L. A. Lompre, G. Mainfray, C. Manus, Multiple-harmonic conversion of 1064 nm radiation in rare gases. J. Phys. B At. Mol. Opt. Phys. 21, L31–L35 (1988).

[13] S. Ghimire, A. D. Dichiara, E. Sistrunk, P. Agostini, L. F. Dimauro, D. A. Reis, Observation of high-order harmonic generation in a bulk crystal. Nat. Phys. 7, 138–141 (2011).

[14] P. Jürgens, B. Liewehr, B. Kruse, C. Peltz, D. Engel, A. Husakou, T. Witting, M. Ivanov, M. J. J. Vrakking, T. Fennel, A. Mermillod-Blondin, Origin of strong-field-induced low-order harmonic generation in amorphous quartz. Nat. Phys. 16, 1035–1039 (2020).

[15] D. Golde, T. Meier, S. W. Koch, High harmonics generated in semiconductor nanostructures by the coupled dynamics of optical inter-and intraband excitations. Phys. Rev. B 77, 075330 (2008).

[16] O. Schubert, M. Hohenleutner, F. Langer, B. Urbanek, C. Lange, U. Huttner, D. Golde, T. Meier, M. Kira, S. W. Koch, R. Huber, Sub-cycle control of terahertz high-harmonic generation by dynamical bloch oscillations. Nat. Photonics 8, 119–123 (2014).

[17] A. Shiner, B. Schmidt, C. Trallero-Herrero, H. J. Wörner, S. Patchkovskii, P. B. Corkum, J.-C. Kieffer, F. Légaré, D. Villeneuve, Probing collective multi-electron dynamics in xenon with high-harmonic spectroscopy. Nat. Phys. 7, 464–467 (2011).

[18] O. Smirnova, Y. Mairesse, S. Patchkovskii, N. Dudovich, D. Villeneuve, P. Corkum, M. Y. Ivanov, High harmonic interferometry of multi-electron dynamics in molecules. Nature 460, 972–977 (2009). 19626004 10.1038/nature08253

[19] P. M. Kraus, B. Mignolet, D. Baykusheva, A. Rupenyan, L. Horný, E. F. Penka, G. Grassi, O. I. Tolstikhin, J. Schneider, F. Jensen, L. B. Madsen, A. D. Bandrauk, F. Remacle, H. J. Wörner, Measurement and laser control of attosecond charge migration in ionized iodoacetylene. Science 350, 790–795 (2015). 26494175 10.1126/science.aab2160

[20] S. Ghimire, D. A. Reis, High-harmonic generation from solids. Nat. Phys. 15, 10–16 (2019).

[21] P. M. Kraus, S. B. Zhang, A. Gijsbertsen, R. R. Lucchese, N. Rohringer, H. J. Wörner, High-harmonic probing of electronic coherence in dynamically aligned molecules. Phys. Rev. Lett. 111, 243005 (2013). 24483654 10.1103/PhysRevLett.111.243005

[22] Z. Wang, H. Park, Y. H. Lai, J. Xu, C. I. Blaga, F. Yang, P. Agostini, L. F. DiMauro, The roles of photo-carrier doping and driving wavelength in high harmonic generation from a semiconductor. Nat. Commun. 8, 1686 (2017). 29162818 10.1038/s41467-017-01899-1 PMC5698516

[23] C. Heide, Y. Kobayashi, A. C. Johnson, F. Liu, T. F. Heinz, D. A. Reis, S. Ghimire, Probing electron-hole coherence in strongly driven 2D materials using high-harmonic generation. Optica 9, 512 (2022).

[24] Y. Wang, F. Iyikanat, X. Bai, X. Hu, S. Das, Y. Dai, Y. Zhang, L. Du, S. Li, H. Lipsanen, F. J. García De Abajo, Z. Sun, Optical control of high-harmonic generation at the atomic thickness. Nano Lett. 22, 8455–8462 (2022). 36305718 10.1021/acs.nanolett.2c02711 PMC9650768

[25] M. R. Bionta, E. Haddad, A. Leblanc, V. Gruson, P. Lassonde, H. Ibrahim, J. Chaillou, N. Émond, M. R. Otto, Á. Jiménez-Galán, R. E. Silva, M. Ivanov, B. J. Siwick, M. Chaker, F. Légaré, Tracking ultrafast solid-state dynamics using high harmonic spectroscopy. Phys. Rev. Res. 3, 023250 (2021).

[26] Y. Cheng, H. Hong, H. Zhao, C. Wu, Y. Pan, C. Liu, Y. Zuo, Z. Zhang, J. Xie, J. Wang, D. Yu, Y. Ye, S. Meng, K. Liu, Ultrafast optical modulation of harmonic generation in two-dimensional materials. Nano Lett. 20, 8053–8058 (2020). 33112622 10.1021/acs.nanolett.0c02972

[27] M. L. S. van der Geest, J. J. de Boer, K. Murzyn, P. Jürgens, B. Ehrler, P. M. Kraus, Transient high-harmonic spectroscopy in an inorganic-organic lead halide perovskite. J. Phys. Chem. Lett. 14, 10810–10818 (2023). 38015825 10.1021/acs.jpclett.3c02588 PMC10711791

[28] P. J. van Essen, Z. Nie, B. de Keijzer, P. M. Kraus, Towards complete all-optical emission control of high-harmonic generation from solids. ACS Photonics 11, 1832–1843 (2024). 38766500 10.1021/acsphotonics.4c00156 PMC11100285

[29] B. de Keijzer, P. J. van Essen, P. M. Kraus, Effect of photoexcitation on high-harmonic generation in semiconductors. J. Opt. Soc. Am. B 41, 1754–1763 (2024).

[30] G. G. Brown, Á. Jiménez-Galán, R. E. F. Silva, M. Ivanov, A real-space perspective on dephasing in solid-state high harmonic generation. arXiv:2210.16889 [physics.optics] (2022).

[31] Y. Wang, Y. Liu, P. Jiang, Y. Gao, H. Yang, L. Y. Peng, Q. Gong, C. Wu, Optical switch of electron-hole and electron-electron collisions in semiconductors. Phys. Rev. B 107, L161301 (2023).

[32] M. Weber, H. von der Emde, M. Leutenegger, P. Gunkel, S. Sambandan, T. A. Khan, J. Keller-Findeisen, V. C. Cordes, S. W. Hell, MINSTED nanoscopy enters the Ångström localization range. Nat. Biotechnol. 41, 569–576 (2023). 36344840 10.1038/s41587-022-01519-4 PMC10110459

[33] E. Betzig, Proposed method for molecular optical imaging. Opt. Lett. 20, 237–239 (1995). 19859146 10.1364/ol.20.000237

[34] E. Betzig, G. H. Patterson, R. Sougrat, O. W. Lindwasser, S. Olenych, J. S. Bonifacino, M. W. Davidson, J. Lippincott-Schwartz, H. F. Hess, Imaging intracellular fluorescent proteins at nanometer resolution. Science 313, 1642–1645 (2006). 16902090 10.1126/science.1127344

[35] M. J. Rust, M. Bates, X. Zhuang, Sub-diffraction-limit imaging by stochastic optical reconstruction microscopy (STORM). Nat. Methods 3, 793–796 (2006). 16896339 10.1038/nmeth929 PMC2700296

[36] A. Sharonov, R. M. Hochstrasser, Wide-field subdiffraction imaging by accumulated binding of diffusing probes. Proc. Natl. Acad. Sci. U.S.A. 103, 18911–18916 (2006). 17142314 10.1073/pnas.0609643104 PMC1748151

[37] S. W. Hell, J. Wichmann, Breaking the diffraction resolution limit by stimulated emission: Stimulated-emission-depletion fluorescence microscopy. Opt. Lett. 19, 780–782 (1994). 19844443 10.1364/ol.19.000780

[38] T. A. Klar, S. Jakobs, M. Dyba, A. Egner, S. W. Hell, Fluorescence microscopy with diffraction resolution barrier broken by stimulated emission. Proc. Natl. Acad. Sci. U.S.A. 97, 8206–8210 (2000). 10899992 10.1073/pnas.97.15.8206 PMC26924

[39] S. W. Hell, M. Kroug, Ground-state-depletion fluorscence microscopy: A concept for breaking the diffraction resolution limit. Appl. Phys. B 60, 495–497 (1995).

[40] M. G. Gustafsson, Nonlinear structured-illumination microscopy: Wide-field fluorescence imaging with theoretically unlimited resolution. Proc. Natl. Acad. Sci. U.S.A. 102, 13081–13086 (2005). 16141335 10.1073/pnas.0406877102 PMC1201569

[41] Z. Nie, L. Guery, E. B. Molinero, P. Juergens, T. J. van den Hooven, Y. Wang, A. Jimenez Galan, P. C. M. Planken, R. E. F. Silva, P. M. Kraus, Following the nonthermal phase transition in niobium dioxide by time-resolved harmonic spectroscopy. Phys. Rev. Lett. 131, 243201 (2023). 38181131 10.1103/PhysRevLett.131.243201

[42] H. Pinhas, O. Wagner, Y. Danan, M. Danino, Z. Zalevsky, M. Sinvani, Plasma dispersion effect based super-resolved imaging in silicon. Opt. Express 26, 25370–25380 (2018). 30469640 10.1364/OE.26.025370

[43] P. Wang, M. N. Slipchenko, J. Mitchell, C. Yang, E. O. Potma, X. Xu, J. X. Cheng, Far-field imaging of non-fluorescent species with subdiffraction resolution. Nat. Photonics 7, 449–453 (2013). 24436725 10.1038/nphoton.2013.97 PMC3891596

[44] H. Kanou, Y. Iketaki, Super-resolution microscope (Japan Patent JP2018120006A, January 2017).

[45] L. Gong, W. Zheng, Y. Ma, Z. Huang, Higher-order coherent anti-Stokes Raman scattering microscopy realizes label-free super-resolution vibrational imaging. Nat. Photonics 14, 115–122 (2020).

[46] D. Raanan, M. S. Song, W. A. Tisdale, D. Oron, Super-resolved second harmonic generation imaging by coherent image scanning microscopy. Appl. Phys. Lett. 120, 071111 (2022).

[47] J. J. Field, K. A. Wernsing, S. R. Domingue, A. M. Motz, K. F. DeLuca, D. H. Levi, J. G. DeLuc, M. D. Young, J. A. Squier, R. A. Bartels, Superresolved multiphoton microscopy with spatial frequency-modulated imaging. Proc. Natl. Acad. Sci. U.S.A. 113, 6605–6610 (2016). 27231219 10.1073/pnas.1602811113 PMC4914181

[48] O. Masihzadeh, P. Schlup, R. A. Bartels, Enhanced spatial resolution in third-harmonic microscopy through polarization switching. Opt. Lett. 34, 1240–1242 (2009). 19370130 10.1364/ol.34.001240

[49] S. B. Penwell, L. D. Ginsberg, R. Noriega, N. S. Ginsberg, Resolving ultrafast exciton migration in organic solids at the nanoscale. Nat. Mater. 16, 1136–1141 (2017). 28920937 10.1038/nmat4975

[50] Z. Yang, C. Ko, S. Ramanathan, Oxide electronics utilizing ultrafast metal-insulator transitions. Annu. Rev. Mat. Res. 41, 337–367 (2011).

[51] S. Kumar, R. S. Williams, Z. Wang, Third-order nanocircuit elements for neuromorphic engineering. Nature 585, 518–523 (2020). 32968256 10.1038/s41586-020-2735-5

[52] S. R. Abbing, F. Campi, F. S. Sajjadian, N. Lin, P. Smorenburg, P. M. Kraus, Divergence control of high-harmonic generation. Phys. Rev. Appl. 13, 054029 (2020).

[53] S. D. Roscam Abbing, F. Campi, A. Zeltsi, P. Smorenburg, P. M. Kraus, Divergence and efficiency optimization in polarization-controlled two-color high-harmonic generation. Sci. Rep. 11, 24253 (2021). 34930994 10.1038/s41598-021-03657-2 PMC8688547

[54] M. Negro, C. Vozzi, K. Kovacs, C. Altucci, R. Velotta, F. Frassetto, L. Poletto, P. Villoresi, S. de Silvestri, V. Tosa, S. Stagira, Gating of high-order harmonics generated by incommensurate two-color mid-IR laser pulses. Laser Phys. Lett. 8, 875–879 (2011).

[55] S. Xu, H. Zhang, J. Yu, Y. Han, Z. Wang, J. Hu, Ultrafast modulation of a high harmonic generation in a bulk ZnO single crystal. Opt. Express 30, 41350–41358 (2022). 36366615 10.1364/OE.462638

[56] V. Westphal, S. W. Hell, Nanoscale resolution in the focal plane of an optical microscope. Phys. Rev. Lett. 94, 143903 (2005). 15904066 10.1103/PhysRevLett.94.143903

[57] S. Koho, G. Tortarolo, M. Castello, T. Deguchi, A. Diaspro, G. Vicidomini, Fourier ring correlation simplifies image restoration in fluorescence microscopy. Nat. Commun. 10, 3103 (2019). 31308370 10.1038/s41467-019-11024-z PMC6629685

[58] M. Van Heel, M. Schatz, Fourier shell correlation threshold criteria. J. Struct. Biol. 151, 250–262 (2005). 16125414 10.1016/j.jsb.2005.05.009

[59] S. D. Roscam Abbing, R. Kolkowski, Z. Y. Zhang, F. Campi, L. Lötgering, A. F. Koenderink, P. M. Kraus, Extreme-ultraviolet shaping and imaging by high-harmonic generation from nanostructured silica. Phys. Rev. Lett. 128, 223902 (2022). 35714263 10.1103/PhysRevLett.128.223902

[60] W. P. Beeker, C. J. Lee, K. J. Boller, P. Groß, C. Cleff, C. Fallnich, H. L. Offerhaus, J. L. Herek, A theoretical investigation of super-resolution CARS imaging via coherent and incoherent saturation of transitions. J. Raman Spectrosc. 42, 1854–1858 (2011).

[61] S. Zhang, J. Shi, H. Zhang, T. Jia, Z. Wang, Z. Sun, Precise control of state-selective excitation in stimulated Raman scattering. Phys. Rev. A 82, 043841 (2010).

[62] K. Sekiguchi, S. Yamaguchi, T. Tahara, Femtosecond time-resolved electronic sum-frequency generation spectroscopy: A new method to investigate ultrafast dynamics at liquid interfaces. J. Chem. Phys. 128, 114715 (2008). 18361609 10.1063/1.2841023

[63] Y. Wang, R. B. Comes, S. Kittiwatanakul, S. A. Wolf, J. Lu, Epitaxial niobium dioxide thin films by reactive-biased target ion beam deposition. J. Vac. Sci. Technol. 33, 021516 (2015).

