# Supplementary material for: Breaking Abbe’s diffraction limit with harmonic deactivation microscopy
Source: Sci Adv. 2024 Nov 13;10(46):eadp3056. doi: 10.1126/sciadv.adp3056 (PMC11559608; doi:10.1126/sciadv.adp3056)
Supplement: Supplementary file 1 — Supplementary Text Figs. S1 to S7 [file sciadv.adp3056_sm.pdf]

Science Advances
AAAS

Supplementary Materials for

Breaking Abbe's diffraction limit with harmonic deactivation microscopy

Kevin Murzyn *et al.*

Corresponding author: Kevin Murzyn, k.murzyn@arcnl.nl; Peter M. Kraus, p.kraus@arcnl.nl

Sci. Adv. **10**, eadp3056 (2024)
DOI: 10.1126/sciadv.adp3056

This PDF file includes:

Supplementary Text
Figs. S1 to S7

Supplementary Text

Experiments

The experimental setup is a modified Mach-Zehnder interferometer (Fig. S1). The arm in the vertical direction is the generating arm and in the horizontal direction is the arm of the deactivation pulses. In the generating arm, an optical parametric amplifier (OPA) converts the wavelength of the fundamental pulse from 800 nm to 1800 nm (NIR pulse). In the deactivation arm, a vortex phase plate is inserted to generate the donut pulse. Three different ways enable the detection of emitted harmonics. A wide field microscope images the point-spread function (PSF). A UV/Vis spectrometer resolves the spectrum in reflection. An avalanche photodiode detects individual pulses for raster scanning imaging.

Resolution limit of HADES

The formula for the resolution limit starts at Abbe's diffraction limit for the NIR excitation laser with wavelength λ_0:

$$\mathrm{d}_0 = \frac{\lambda_0}{2 * \mathrm{NA}}. \quad \text{(S 1)}$$

Where the d_0 is the minimum distance of two point sources. For focusing a laser pulse the effective NA can be calculated as:

$$\mathrm{NA} = \frac{\pi * \mathrm{w}_0}{4 * \mathrm{f}}, \quad \text{(S 2)}$$

With the pulse waist w_0 of the collimated pulse and the focus length f of the lens. We assume perturbative non-linear optics therefore the initial Gaussian-shaped pulse intensity of the n-th harmonic can be described as:

$$\mathrm{I}_{\mathrm{nth-HG}}(x,y) \sim I_{NIR}^{n}(x,y) \; = \exp\left(-\frac{x^2+y^2}{(d_0/\sqrt{n})^2}\right), \quad \text{(S 3)}$$

Extracting the pulse waist leads to the resolution of the n-th harmonic as:

$$d = \frac{\lambda_0}{2 * \mathrm{NA} * \sqrt{\mathrm{n}}}. \quad \text{(S 4)}$$

The introduction of the resolution is based on the phenomenological observation that in the simulation the improvement will increase if the peak fluence increases. Therefore, we adapt the formula in (46) to be:

$$d_{HADES} = \frac{\lambda_0}{2 * \mathrm{NA} * \sqrt{\mathrm{n}}} * \frac{1}{\sqrt{1+\zeta}}, \quad \text{(S 5)}$$

with the saturation level ζ.

PSF Model for one and two-dimension

Two models are introduced for estimating the point-spread function (PSF) reduction in one and two dimensions (Fig. S2, S3). For one dimension a Gaussian PSF of the NIR excitation pulse was assumed which then translates to a Gaussian distribution of the THG as shown above. We approximated the intensity profile of the one-dimensional donut beam with a $\cos^2$ function. The experimental results of the deactivation from Fig. 2 are then interpolated into the donut profile.

Multiplying the deactivation profile with the Gaussian distribution leads to a narrower PSF.
For the two-dimensional case, the experimental observed donut intensity distribution was used to interpolate the deactivation profile. The uneven surface of the sample leads to an underestimation of the achievable PSF.

Convolution of HADES

The images obtained by HADES were convolved with a 2D Gaussian distribution Fig. S4. The Gaussian distribution had to be made asymmetric and rotated. To minimize edge effect the convolution was carried out in accordance with the convolution theorem. The following formula was used for the Gaussian distribution in the frequency space:

$$f(x, y) = \frac{1}{2\pi\sigma_x\sigma_y} exp(-(ax^2 + bxy + cy^2), \tag{S 6}$$

with

$$a = \frac{\cos^2(\theta)}{2\sigma_x} + \frac{\sin^2(\theta)}{2\sigma_y}, \tag{S 7}$$

$$b = -\frac{sin(2\theta)}{4\sigma_x} + \frac{sin(2\theta)}{4\sigma_y}, \tag{S 8}$$

$$c = \frac{\sin^2(\theta)}{2\sigma_x} + \frac{\cos^2(\theta)}{2\sigma_y}. \tag{S 9}$$

The parameters to obtain the resulting convolution are $\sigma_x = 0.08\ \mu m^{-1}$, $\sigma_y = 0.13\ \mu m^{-1}$ and $\theta = 45°$. The multiplication of the Fourier transformed picture with the Gaussian was inverse Fourier transformed to get the convolution. As harmonic generation is generally a coherent process, in principle any convolution needs to take into account the phase of the emitted light. We cannot access the phase in our current experiments. However, the sample we are looking at is mostly governed by amplitude variations, consisting of regions with sample (bright emission) and no sample (no emission). Therefore, assuming a flat phase, which is equivalent to an intensity cross correlation as performed above, is a fair assumption.

Fourier ring correlation

To quantify the resolution improvement, we use the single image Fourier ring correlation (FRC) algorithm by [57] on the images in Fig 4b) and 4c). The Fourier transform of each image was masked at the axis to suppress scanning artifacts (Fig. S5a,b). The colorbars in these images are shown on a logarithmic scale. For the single image FRC (Fig. S5c), the pixels get subdivided into two pairs of subimages. A FRC is performed, and the resolution is determined at the 1/7 threshold. The reduced range in frequency space is explained by this subdivision a recalibration is needed as descirebed in [57].

Light microscopy images

The bright-field microscopy (Fig. S6a) images were taken with an effective magnification of 80. The dark-field microscopy images were taken with an effective magnification of 50. The position was traced back through the absolute coordinates of the stage in the Mach-Zehnder interferometer in combination with markers on the sample. The Darkfield microscopy (Fig. S6b) images show a roughening of the surface of the sample.

Electron Microscopy images

In the electron microscopy images (Fig. S7), the nanostructure from the laser damage becomes visible. This nanostructure enhances the third harmonic generation of the thin film. The deep “L” shaped trench shows a complete ablation of the NbO_2 film.

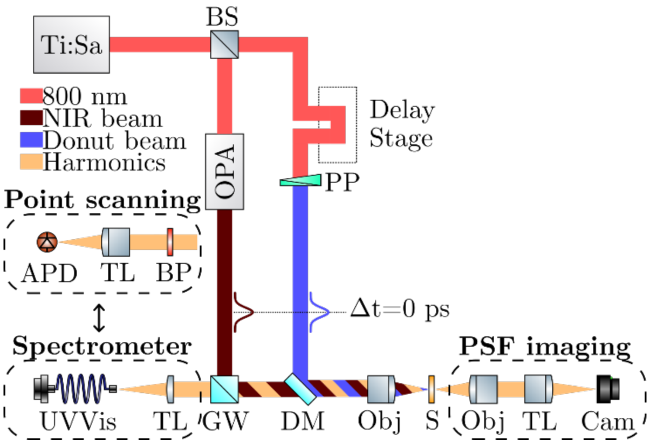

Fig. S1. Experimental setup

The experimental setup is a modified Mach-Zehnder interferometer. In the generating arm, an optical parametric amplifier (OPA) converts the wavelength of the fundamental beam from 800 nm to 1800 nm (NIR beam). In the deactivation arm, a spiral phase plate is inserted to generate the donut beam. Three different ways enable the detection of emitted harmonics. A wide field microscope images the point-spread function (PSF). A UV/Vis spectrometer resolves the spectrum in reflection. An avalanche photodiode detects individual pulses for raster scanning imaging. For more details refer to the main text. BS, beam splitter; OPA, optical parametric amplifier; PP, spiral phase plate; GW, glass wedge; DM, dichroic mirror; Obj, objective lens; S, sample; TL, tube lens; Cam, camera; BP, bandpass filter; UVVis, Fiber spectrometer; APD, avalanche photodiode.

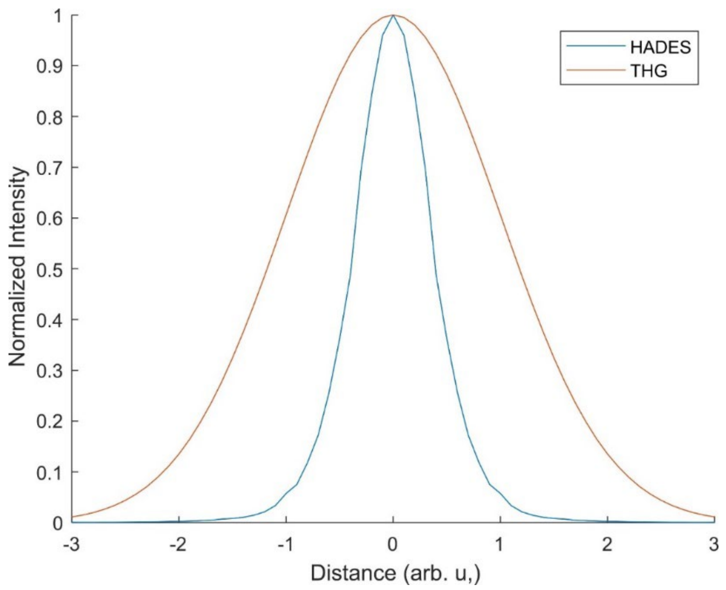

Fig. S2. One-dimensional model of point-spread function

Assumed Gaussian intensity distribution for third harmonic generation (orange). This is combined with a deactivation profile which is assumed to be of the form $\cos^2(x)$, where x is the distance. The deactivation is taken from the normalized deactivation in Fig. 2 of the main text. The peak fluence is assumed to be 25 mJ/cm². The resulting Gaussian peak of the harmonic deactivation microscopy (HADES) is smaller by a factor of three.

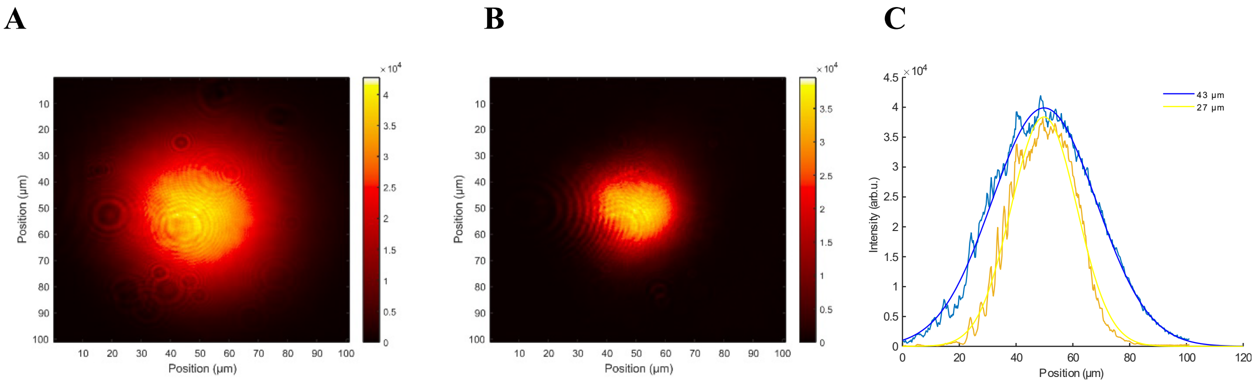

Fig. S3. Two-dimensional model

Two-dimensional (2D) model of the deactivation PSF based on experimental data. The original PSF of the third harmonic (a) gets deactivated by the donut beam (inset Fig. 3c). Assuming a peak fluence of 25 mJ/cm^2 the resulting PSF is shown (b). Lineouts of the PSF are shown in c) with the FWHM of the 1D fits of THG and HADES respectively. The 2D model shows an underestimation of the experimentally reached deactivation. This can partially be backtracked to the diffraction pattern from the imaging path of the donut beam. These diffraction patterns lead to a wrong estimation of the peak fluence position, and therefore an underestimation of the fluence profile.

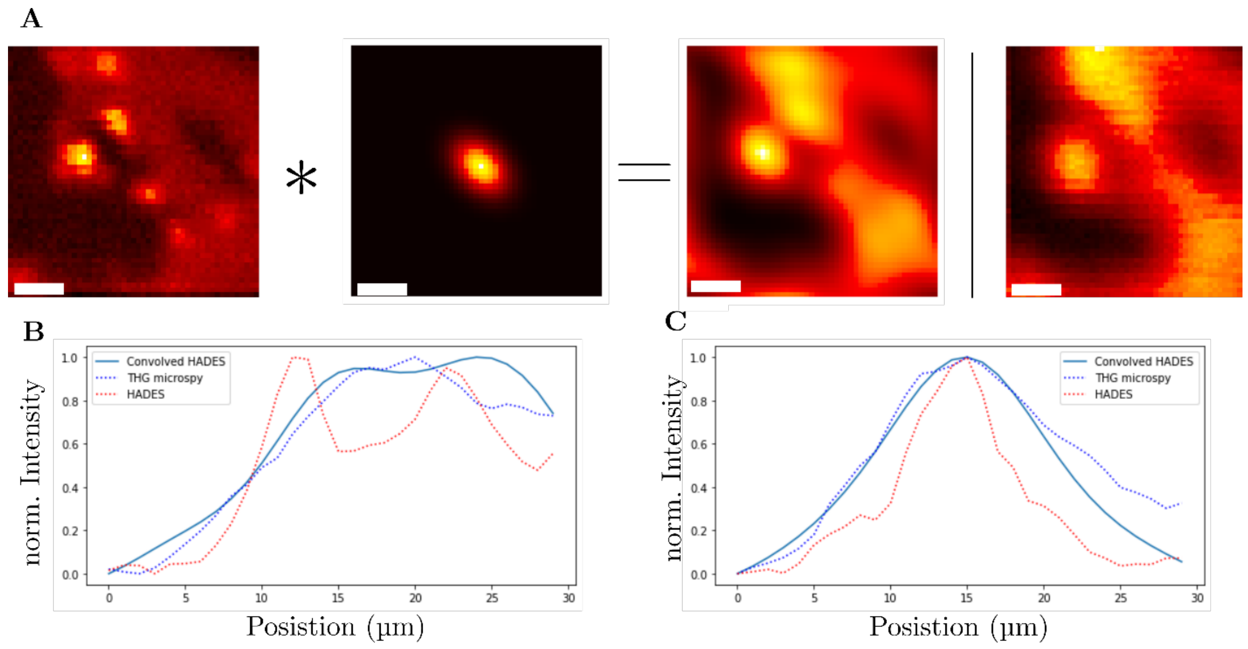

Fig. S4. Convolutions of HADES images

a) The whole image taken by HADES is convolved with an asymmetric and rotated Gaussian distribution. The resulting image resembles the image taken by third-harmonic generation (THG) microscopy (far right). b) Dotted profile as shown in Fig 4. of the main text. The solid line shows a convolution of the HADES profile with a Gaussian. Only on the edges of the profile lines does the convolution show bigger differences to the THG profile. c) Dashed profile as shown in Fig. 4 in the main text. The solid line shows the convolution of the HADES profile. At the right edge, the convolved lineout shows a larger difference to the THG profile due to the missing 2D information, which contains a blurred edge in the THG image but not in the HADES image.

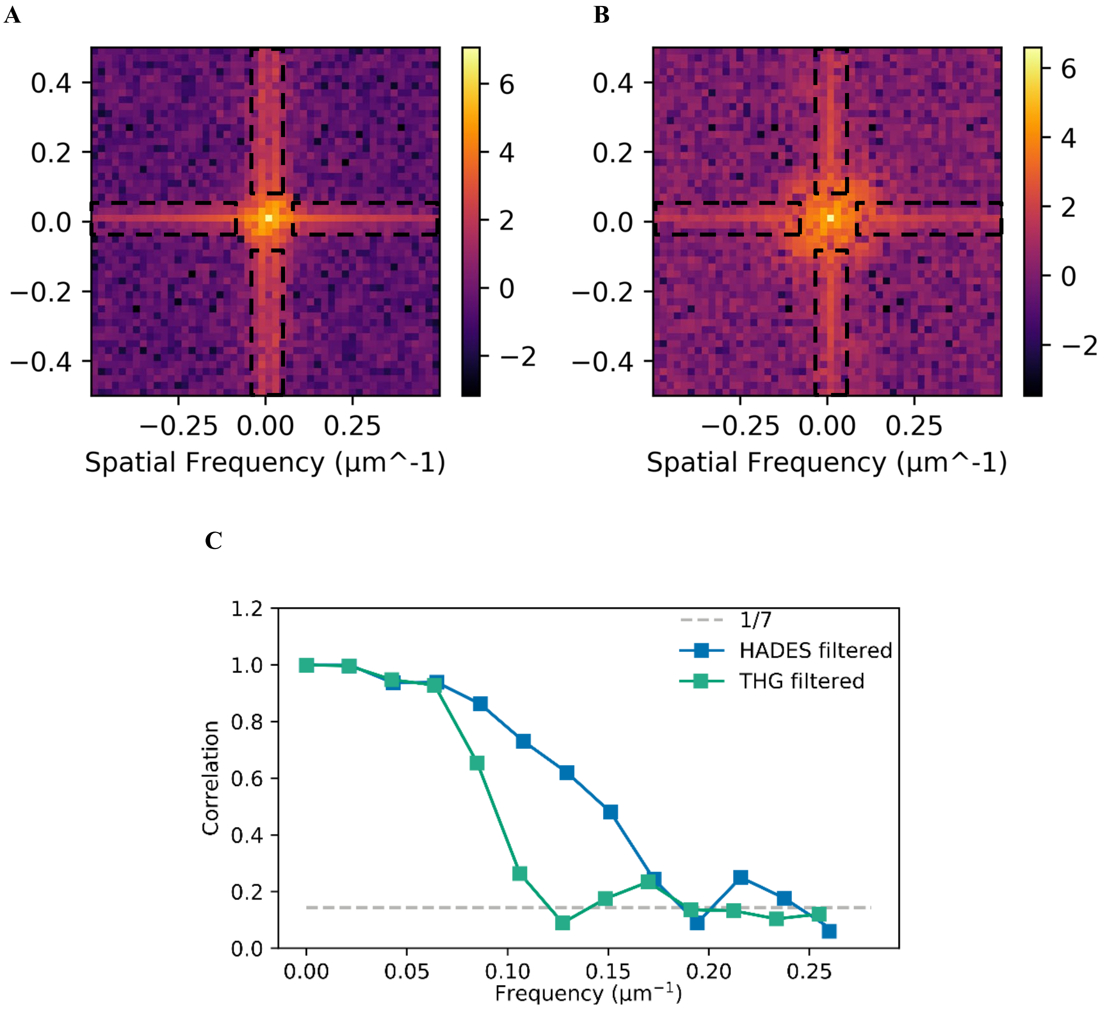

Fig. S5. Fourier ring correlation

Fourier transforms of A) the THG image and B) the HADES image shown in Fig. 4B and Fig. 4C with logarithmic colorbar. For the one-image fourier ring correlation (FRC) introduced in [57] to work the artifacts along the axis were masked (black boxes). C) Result of the one image FRC showing a higher correlation for HADES at larger spatial frequencies and a later crossing of the 1/7-threshold.

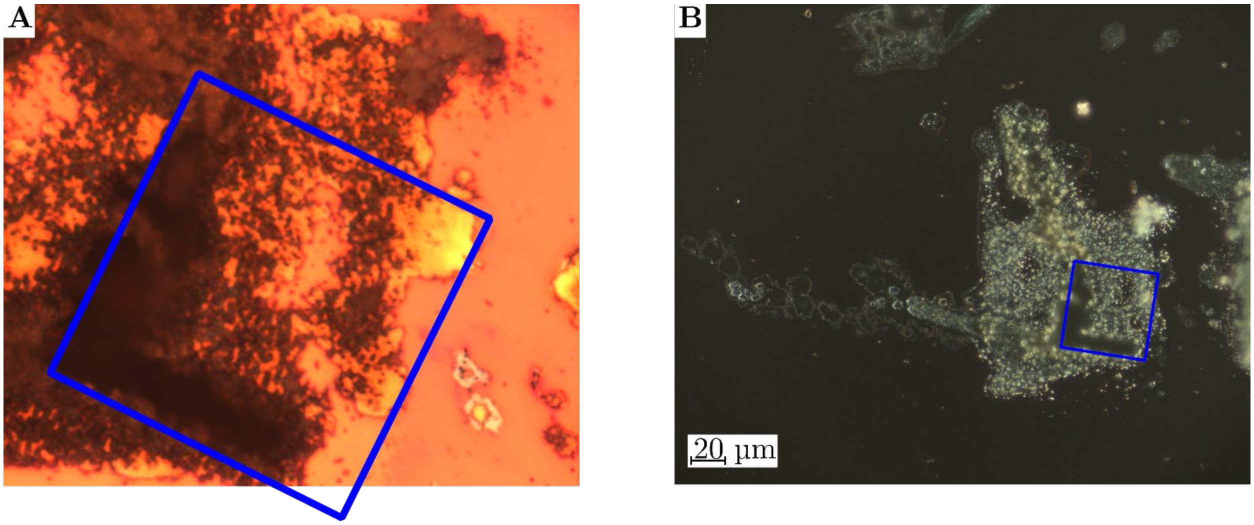

Fig. S6. Full-field microscopy images

Bright-field (a) and dark-field (b) microscopy images of the area images in Fig 4. In the dark field an increased surface roughness of the image can be seen. This indicates the thermal damage of the thin film. The “L”-shaped area is a complete ablation of the thin film.

A B

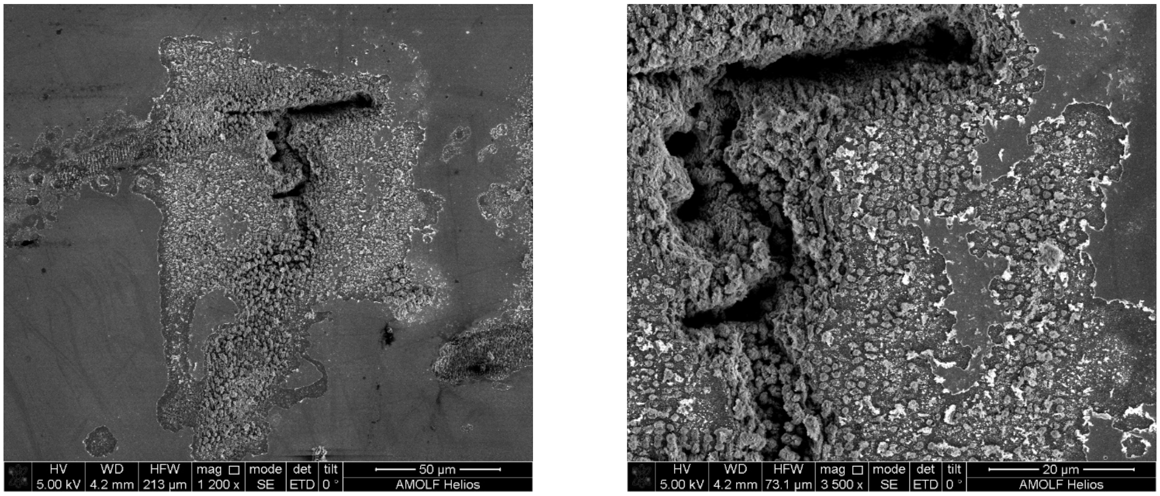

Fig. S7. Scanning electron microscopy images

a) Overview of the scanned area that got imaged with THG and HADES. Compared to the scanning and full-field images these images are flipped along the horizontal axis. b) Zoomed-in version of the scanned images shown before. The nanostructures on the surface are visible. It can be the reason for the enhancement of the THG at certain positions.
